# A Genome-Scale Metabolic Model of Marine Heterotroph Vibrio splendidus Strain 1A01

**DOI:** 10.1128/msystems.00377-22

**Published:** 2023-02-28

**Authors:** Arion Iffland-Stettner, Hiroyuki Okano, Matti Gralka, Ghita Guessous, Kapil Amarnath, Otto X. Cordero, Terence Hwa, Sebastian Bonhoeffer

**Affiliations:** a Institute of Integrative Biology, ETH Zurich, Zurich, Switzerland; b Department of Physics, University of California at San Diego, La Jolla, California, USA; c Department of Civil and Environmental Engineering, Massachusetts Institute of Technology, Cambridge, Massachusetts, USA; University of British Columbia

**Keywords:** metabolic modeling

## Abstract

While Vibrio splendidus is best known as an opportunistic pathogen in oysters, Vibrio splendidus strain 1A01 was first identified as an early colonizer of synthetic chitin particles incubated in seawater. To gain a better understanding of its metabolism, a genome-scale metabolic model (GSMM) of V. splendidus 1A01 was reconstructed. GSMMs enable us to simulate all metabolic reactions in a bacterial cell using flux balance analysis. A draft model was built using an automated pipeline from BioCyc. Manual curation was then performed based on experimental data, in part by gap-filling metabolic pathways and tailoring the model’s biomass reaction to V. splendidus 1A01. The challenges of building a metabolic model for a marine microorganism like V. splendidus 1A01 are described.

**IMPORTANCE** A genome-scale metabolic model of V. splendidus 1A01 was reconstructed in this work. We offer solutions to the technical problems associated with model reconstruction for a marine bacterial strain like V. splendidus 1A01, which arise largely from the high salt concentration found in both seawater and culture media that simulate seawater.

## INTRODUCTION

The heterotrophic, Gram-negative species Vibrio splendidus is found ubiquitously in the ocean, both in close association with marine animals (like bivalves [1–3]) and as an “environmental” microorganism in marine microbial communities (4, 5). When associated with marine animals, V. splendidus, as a pathogen, induces vibriosis (6) and is of relevance to the aquaculture industry, causing outbreaks in hatcheries around the world, at great economic cost (2). But it is as an environmental microorganism, with an ecological role to play in establishing microbial communities on ocean particles, that the V. splendidus 1A01 strain was first isolated (5). V. splendidus 1A01 was identified as an early colonizer of synthetic chitin particles incubated in seawater samples, secreting enzymes that break down chitin, thereby laying the groundwork for microbial community assembly (5).

Genome-scale metabolic models (GSMMs) have proven to be powerful tools in systems biology for simulating the metabolism of bacteria such as Escherichia coli (7–9). Mathematically, a GSMM is comprised of a stoichiometric matrix encoding all of the reactions in a cell, in addition to exchange fluxes with the environment (7, 10). The model also includes a biomass reaction that converts, in experimentally measured proportions, the building blocks of a cell (e.g., nucleic acids, amino acids, vitamins, and cofactors) into biomass (7, 10). Finally, upper and lower bounds are imposed on the permissible flux through every reaction (7, 10). Thus, the model captures the metabolic capabilities of a microorganism for growth conditions where measurements are available or can be extrapolated.

Constraint-based computational methods like flux balance analysis (FBA) can be used to simulate the distribution of fluxes through a whole-cell metabolic network, when supplied with an objective function and constraints (7, 10). Often, the objective function being optimized under steady-state exponential growth is biomass production (7, 10), while a constraint can be the measured carbon uptake rate (7, 10). If so, FBA will calculate the optimal flux through the biomass reaction of a cell given this constraint. By virtue of its implementation through linear programming, FBA is computationally inexpensive (10). Beyond calculating optimal growth rates, FBA has been successfully applied in the context of metabolic engineering (11–13), identifying drug targets (14–16), studying the properties of metabolic networks (17), simulating bacterial growth in three dimensions (18), and predicting cross-feeding interactions within microbial communities (19–21). Finally, metabolic models can be integrated with a variety of omics data, including metabolomics (22–24), transcriptomics (25–27), and proteomics (26, 28).

**FIG 1:**
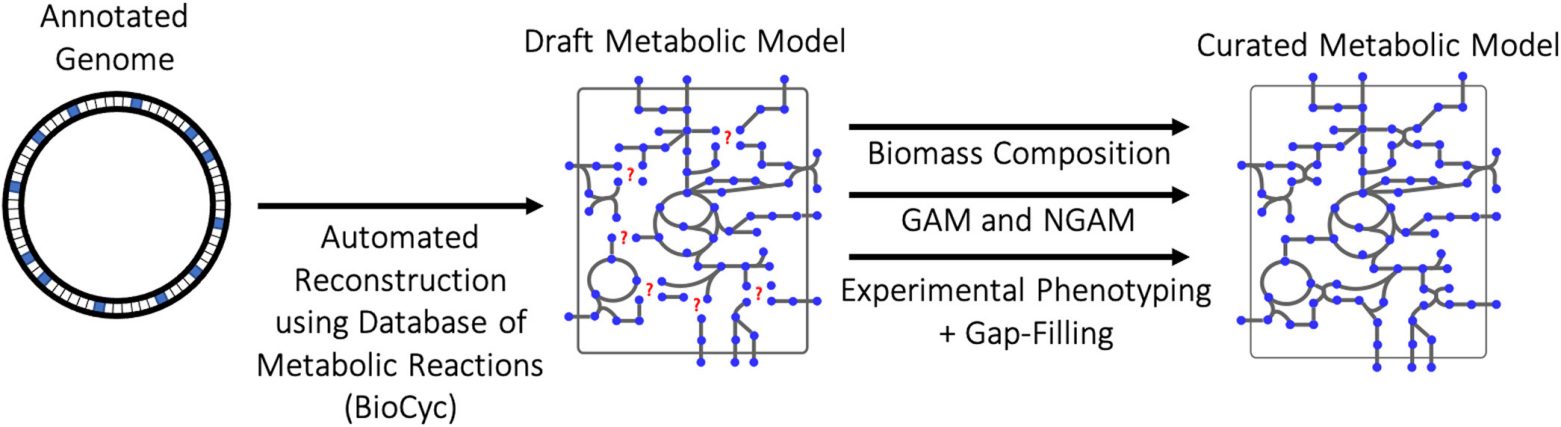
Computational pipeline for building the metabolic model of V. splendidus 1A01.

To facilitate the study of V. splendidus 1A01, a GSMM was reconstructed, which is the first one for a Vibrio splendidus strain (Fig. 1). Reconstruction began by feeding the annotated genome of V. splendidus 1A01 into an automated pipeline from BioCyc (a database of metabolic reactions [29]) to obtain a draft metabolic model. Limited by the well-known incompleteness of genomic annotation (recent estimates of the average annotation completeness for bacterial genomes range from 52% to 79%, depending on the annotation method [30]), the draft metabolic model had to undergo extensive manual curation. First, based on the growth of V. splendidus 1A01 on a wide variety of carbon sources, metabolic pathways were gap-filled (31). Second, the proportions in which cellular building blocks are converted into V. splendidus 1A01 biomass were experimentally measured to curate the biomass reaction of the model. Third, the growth-associated maintenance (GAM) (32) and non-growth-associated maintenance (NGAM) (32) of V. splendidus 1A01 were also measured. The curated model was then quantitatively validated, using still more experimental data. Along the way, building a model for a marine microbe like V. splendidus 1A01 presented a number of technical challenges, which are addressed in the Discussion.

## RESULTS

Due to scientific knowledge gaps with regard to protein function and gene-to-protein mapping, genome annotations are, in general, incomplete (33). Since draft models are automatically reconstructed from genome annotations (32), the shortcomings of genome annotations propagate directly to draft models, which display missing reactions in many metabolic pathways (31). The process of restoring these missing reactions, and obtaining a functional metabolic model, is called gap-filling (31), and it demands phenotypic data (34). In order to test its metabolic capabilities, V. splendidus 1A01 was first cultured on 78 carbon sources for 10 days to obtain a coarse growth phenotype (see Fig. S4 in the supplemental material). Out of these 78 sources, V. splendidus 1A01 grew successfully on 35 carbon sources (Table 1). After gap-filling the relevant metabolic pathways (see Materials and Methods), the model yielded growth on all 35 carbon sources (Table 1). Out of the 43 carbon sources V. splendidus 1A01 failed to grow on, 10 of them enabled growth according to the model (Table 1), provided that, in the model, they freely diffused through the inner membrane of the cell (i.e., they did not require designated transporters to enter the cytoplasm). If, on the contrary, they were barred from freely diffusing through the cell’s inner membrane (i.e., if they required designated transporters to enter the cytoplasm), then only 4 substrates out of 43 enabled growth in the model (Table 1). The discrepancy may be due to the lack of expression of these transporters under the conditions studied.

**TABLE 1:** Experimental phenotyping

Substrate
Enabled growth *in vivo* and *in silico*^*a*^
Acetic acid
Aspartic acid
Citrate
d-Alanine
d-Cellobiose
d-Fructose
d-Galactose
d-Glucosamine
d-Glucose
d-Glucuronic acid
d-Mannose
d-Melibiose
Fumaric acid
GlcNAc
Gluconate
Glutamic acid
Glycerol
Glycine
Histidine
l-Arginine
l-Asparagine
l-Glutamine
l-Lactic acid
l-Proline
l-Serine
l-Threonine
Lactose
Malic acid
Maltose
Mannitol
Oxaloacetic acid
Propionic acid
Pyruvic acid
Succinic acid
Taurine
Enabled growth neither *in vivo* nor *in silico*^*b*^
Arabinose
Beta-alanine
Butyrate
Cystine
d-Galactosamine
Dulcitol
GalNAc
Glycolate
Isoleucine
l-Cysteine
l-Fucose
l-Lysine
l-Lyxose
l-Rhamnose
l-Sorbose
Lactulose
Leucine
*m*-Inositol
Maleic acid
Maltitol
Methionine
PABA
PHBA
Propanol
Raffinose
Sarcosine
Sucrose
Urea
Valeric acid
Valine
Xylitol
Xylose
Enabled growth *in silico* but not *in vivo*^*c*^
Acetaldehyde
Adenine
Ethanol
Ethylene glycol
Formate*
Glyoxylic acid
Methanol
Norvaline
Phenylalanine*
Sorbitol*
Tyrosine*

a The 35 carbon sources V. splendidus 1A01 can grow on, both *in vivo* and *in silico*.

b The 43 carbon sources V. splendidus 1A01 failed to grow on, both *in vivo* and *in silico.*

c The 10 carbon sources V. splendidus 1A01 grew on *in silico* but failed to grow on *in vivo*. The 4 carbon sources that enabled growth *in silico* when diffusing freely into the periplasm but not into the cytoplasm are indicated by asterisks. Of these 4 carbon sources, sorbitol is the only one that did not yield growth when diffusing directly into the cytoplasm (without active transport) because its metabolism requires a phosphorylation reaction catalyzed by a transporter.

10.1128/msystems.00377-22.4 FIG S4 Experimental phenotyping. The full growth curves of V. splendidus 1A01 on all 78 carbon sources. The replicates are technical (same original culture, separate wells) and biological (separate original cultures). Download FIG S4, PDF file, 0.6 MB. Copyright © 2023 Iffland-Stettner et al. 2023 Iffland-Stettner et al. https://creativecommons.org/licenses/by/4.0/ This content is distributed under the terms of the Creative Commons Attribution 4.0 International license.

Following gap-filling, the model contained 1,867 reactions and 1,565 metabolites (Table 2). To further characterize the model, every reaction was assigned, if possible, to a metabolic pathway in BioCyc and every metabolic pathway to a broad functional category. Figure S5 in the supplemental material shows the relative distribution of these broad functional categories across all assigned reactions.

**TABLE 2:** Overview of the V. splendidus 1A01 model

Parameter	*n*
Total reactions	1,867
Internal reactions	1,392
Transport reactions	409
Exchange reactions	66
Total metabolites	1,565
Intracellular metabolites	1,287
Periplasmic metabolites	210
Extracellular metabolites	68

10.1128/msystems.00377-22.5 FIG S5 Distribution of pathway categories in the V. splendidus 1A01 model. The different shades of blue and purple on the left side of the pie chart correspond to biosynthesis categories (e.g., biosynthesis of nucleotides). Download FIG S5, PDF file, 0.03 MB. Copyright © 2023 Iffland-Stettner et al. 2023 Iffland-Stettner et al. https://creativecommons.org/licenses/by/4.0/ This content is distributed under the terms of the Creative Commons Attribution 4.0 International license.

While enzyme-catalyzed reactions make up the bulk of a metabolic model, it also contains a biomass reaction that converts cellular building blocks (such as amino acids and nucleotides) into biomass, in experimentally measured proportions (32). Quantifying these proportions is a vital part of model curation. Instead of measuring the exact concentration of every chemical compound to be found in biomass, it is both sufficient (for modeling purposes) and convenient (experiment-wise) to measure the overall macromolecular composition and to fill the leftover knowledge gaps with data from the better-studied E. coli, which, like V. splendidus 1A01, is a Gram-negative gammaproteobacterium (35). With regard to the biomass coefficients supplied by E. coli, a sensitivity analysis (described in Materials and Methods) was carried out and showed the performance of the model to be robust against slight deviations from the exact values in E. coli (see Fig. S3 in the supplemental material).

10.1128/msystems.00377-22.3 FIG S3 Sensitivity analysis to deviations from E. coli in the biomass reaction of the V. splendidus 1A01 model. Download FIG S3, PDF file, 0.02 MB. Copyright © 2023 Iffland-Stettner et al. 2023 Iffland-Stettner et al. https://creativecommons.org/licenses/by/4.0/ This content is distributed under the terms of the Creative Commons Attribution 4.0 International license.

**FIG 2:**
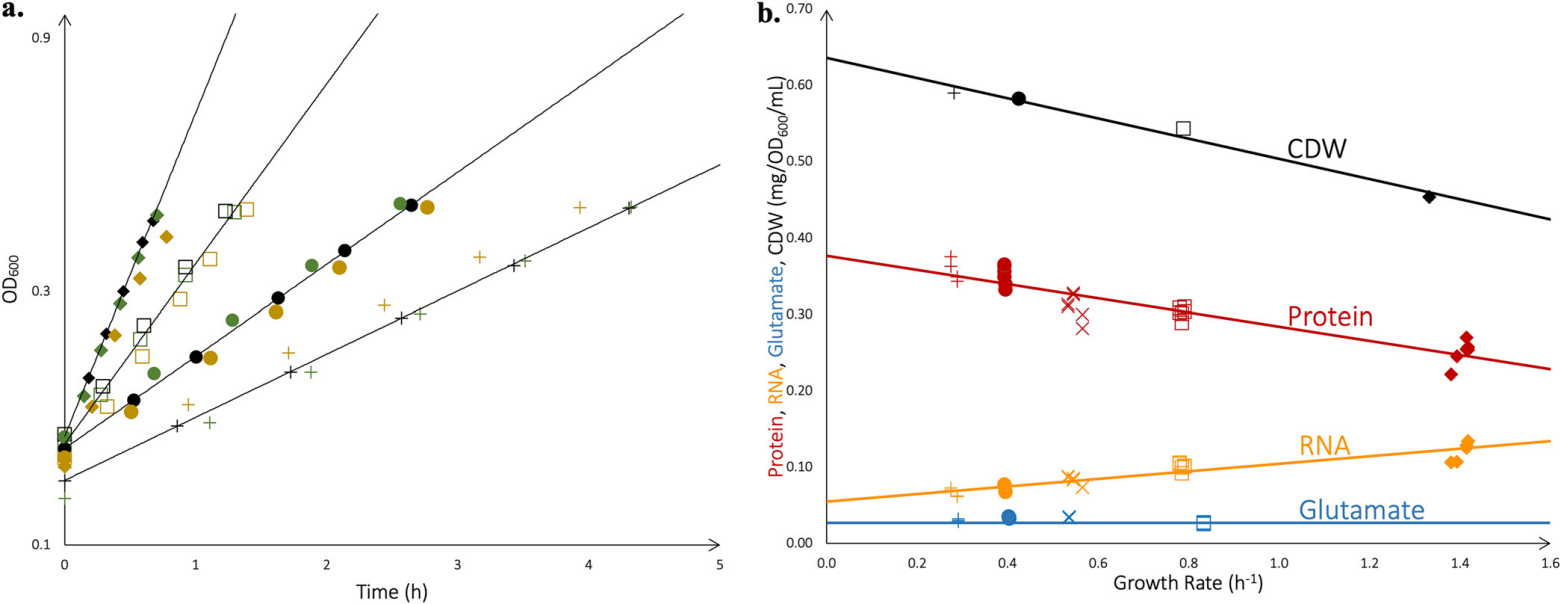
Determination of macromolecular composition during exponential growth. V. splendidus 1A01 was grown in marine broth (♦) and in minimal medium on glucose (□), GlcN (×), glycerol (**●**), and galactose (+), following the protocol described in the Materials and Methods to ensure that each culture was in exponential growth. (a) OD_600_ was recorded during exponential growth, and linear regression was used on these data to determine growth rates. Three replicates (indicated by different colors) are shown for each growth medium. The growth rates obtained were 1.38 ± 0.05 h^−1^ (marine broth), 0.79 ± 0.01 h^−1^ (glucose), 0.40 ± 0.01 h^−1^ (glycerol), and 0.28 ± 0.01 h^−1^ (galactose), with standard deviations computed based on the replicates. The average growth rate for each medium is indicated by a line. (b) For each replicate during exponential growth, the total content of RNA (orange), protein (red), and glutamate (blue) was determined per OD_600_*mL of culture and plotted against growth rate. For four of the growth media, CDW (black) of the culture was also obtained, as described in the Materials and Methods. The lines are the best linear fits to the respective data sets (with y = 0.05x + 0.05 for the total RNA content, y = −0.09x + 0.38 for the total protein content, and y = −0.13x + 0.64 for CDW) with the exception of the glutamate pool, where no clear growth-rate dependence can be seen from the data and the blue line indicates the average value measured for growth on glucose (5.0%), which is similar to the average measured for the four used growth media (5.3%).

Protein, RNA, and osmolytes were expected to dominate the macromolecular composition of V. splendidus 1A01 and were quantified accordingly. Since they are known to be generically growth-rate dependent (36), we measured the content of RNA and proteins in a culture volume of V. splendidus 1A01 grown on a variety of carbon sources, covering a spectrum of growth rates (representative growth curves are shown in Fig. 2a). The results were obtained per optical density at 600 nm (OD_600_)*mL of culture volume, shown in orange (RNA) and red (protein) in Fig. 2b. To make them biologically meaningful, we additionally measured the cell dry weight (CDW) per OD_600_*mL of culture volume for each growth medium. This process required the development of a new protocol as described in the Materials and Methods, and the results are displayed in black in Fig. 2b. Our data show that the RNA, protein, and CDW composition of an exponentially growing culture of V. splendidus 1A01 all vary linearly as a function of growth rate, as indicated by the best-fit lines in Fig. 2b. These linear growth rate dependencies are used in our growth-rate dependent formulation of FBA (see Materials and Methods). Additionally, we found very high glutamate pools in V. splendidus 1A01, amounting to 5 to 6% of CDW (blue symbols in Fig. 2b). (In comparison, the pool of glutamine, which is closely related to glutamate metabolically, was found to be over 10× lower, as shown in Table S2 online at http://github.com/ArionIfflandStettner/1A01). Such large amounts of glutamate suggest it is the major osmolyte of V. splendidus 1A01 (see Discussion). As the data do not indicate a clear growth-rate dependence for glutamate content, in the biomass reaction of the model, we used the average glutamate content on glucose (5%), which is very close to the average glutamate content measured across all media tested (5.3%). Together, the trends in Fig. 2b define the major components of the GR-dependent biomass composition of V. splendidus 1A01, as determined by our experiments.

**FIG 3:**
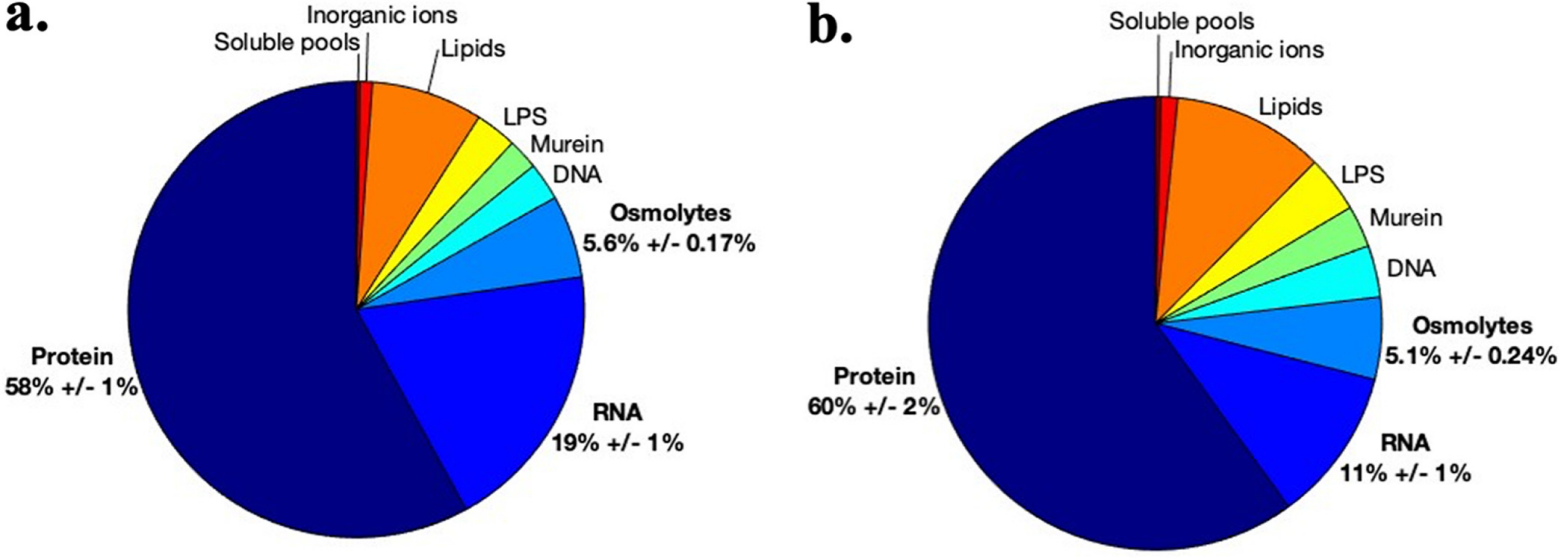
Macromolecular biomass composition of V. splendidus 1A01 growing on glucose (a) and galactose (b). Bold text shows the three components of biomass (protein, RNA, and osmolytes) for which experimental data were collected (together with percentage values of total CDW ± one standard deviation). Combined, they make up 83% of the CDW of V. splendidus 1A01 (on glucose). The remaining 17% of its biomass (DNA, LPS, lipids, murein, inorganic ions, and soluble pools) reflect the E. coli model iAF1260 (37).

The biomass composition of V. splendidus 1A01 growing on glucose (at a growth rate of 0.79 h^−1^) is shown in Fig. 3a. (In Fig. S6 in the supplemental material is shown the biomass composition of E. coli, which is qualitatively similar.) For comparison, Fig. 3b shows the biomass composition of V. splendidus 1A01 growing on galactose, at the lower growth rate of 0.29 h^−1^. As expected from Fig. 2b, due to the higher growth rate associated with glucose compared with that associated with galactose, RNA accounts for a visibly greater percentage of CDW in Fig. 3a than that in Fig. 3b. We selected the biomass composition of V. splendidus 1A01 growing on glucose to build the biomass reaction of the model (which can be found in Table S3 at http://github.com/ArionIfflandStettner/1A01).

10.1128/msystems.00377-22.6 FIG S6 Macromolecular biomass composition of E. coli iAF1260 (from reference 37). Download FIG S6, PDF file, 0.02 MB. Copyright © 2023 Iffland-Stettner et al. 2023 Iffland-Stettner et al. https://creativecommons.org/licenses/by/4.0/ This content is distributed under the terms of the Creative Commons Attribution 4.0 International license.

**FIG 4:**
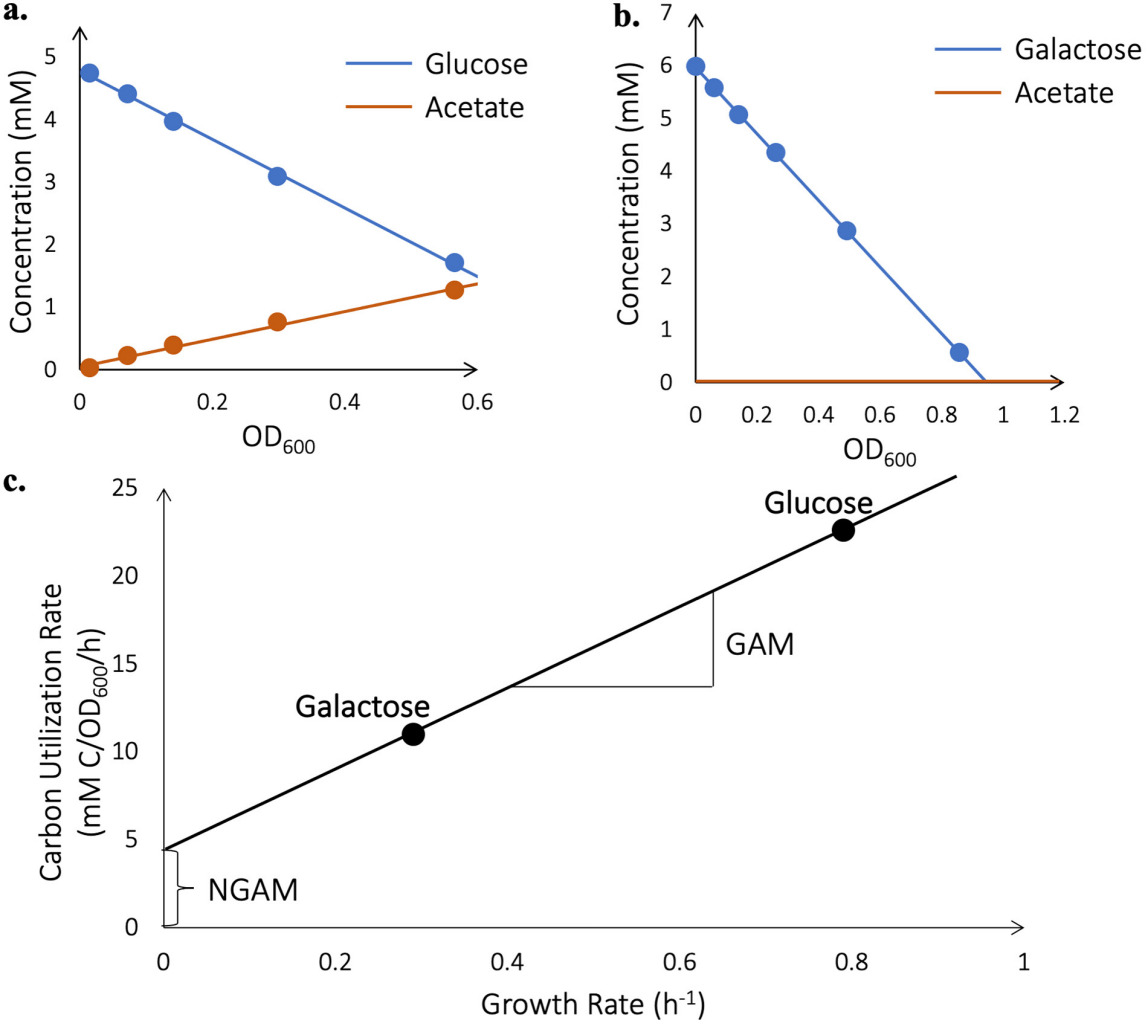
GAM and NGAM. (a and b) Shown in red is acetate accumulation, and shown in blue is glucose or galactose depletion for a single batch culture of V. splendidus 1A01 (no replicates were performed). Acetate is not secreted when V. splendidus 1A01 is grown on galactose. The consumption yields of growth on glucose and galactose, together with the excretion yields of acetate, correspond to the slopes of blue and red plots, respectively. (c) The carbon utilization rate then corresponds to the difference between consumption and excretion yields, multiplied by the growth rate on each carbon source. The GAM is calculated based on the slope of the line, the NGAM is calculated based on its y-intercept. Regarding the units, mM C corresponds to mM of carbon atoms in the substrates.

The third and final step of model curation is to estimate the NGAM, which corresponds to the amount of energy the cell consumes just to survive (32) (by, for example, maintaining the integrity of its membrane), and the GAM, which corresponds to the amount of energy the cell consumes to produce biomass (32) (by, for example, polymerizing amino acids into protein). To estimate these values, V. splendidus 1A01 was grown in batch culture on glucose and on galactose as the sole carbon substrate. The optical density, concentration of the carbon substrate, and concentration of the excreted acetate were measured at various time intervals throughout exponential batch-culture growth. From the temporal dependence of the optical density measurements, growth rates were deduced (0.79 h^−1^ for glucose and 0.29 h^−1^ for galactose). Plotting the concentration of the carbon substrate or the excreted acetate against optical densities, we obtained the consumption yields of growth on glucose and on galactose, together with the excretion yield of acetate, as the slopes of the respective plots (Fig. 4a and b). The difference of the consumption and excretion yields (in units of carbon monomer per OD_600_) was then multiplied by the growth rate to obtain the carbon utilization rate (i.e., the rate at which carbon monomers are either incorporated into biomass or used for energy biogenesis) in each growth medium. The resulting carbon utilization rates were plotted in Fig. 4c against the corresponding growth rates. The y-intercept of this plot, 4.22 mM-C/OD_600_/h, represents the rate of carbon utilization by V. splendidus 1A01 at zero growth rate, when carbon is utilized, not to produce biomass, but strictly to generate energy for cell “maintenance.” Using FBA, we found the maximal rate of ATP production given this carbon utilization rate and zero flux through the biomass reaction. The NGAM corresponds to this maximal rate of ATP production in the absence of growth and shows up in the model as the minimum allowable flux (12.8 mmol/gCDW/h) through an ATP hydrolysis reaction, which must be satisfied under all conditions (32). However, the GAM (measured at 15.8 mmol/gCDW) features in the biomass reaction of the model (32) and reflects the slope of the line in Fig. 4c (23.3 mM-C/OD_600_). For comparison, the E. coli model iAF1260 (37) has an NGAM of 8.39 mmol/gCDW/h (versus 12.8 mmol/gCDW/h in 1A01) and a GAM of 59.81 mmol/gCDW (versus 15.8 mmol/gCDW in 1A01).

As mentioned above, we selected the biomass composition of V. splendidus 1A01 grown on glucose to build the biomass reaction of the model (see Table S3 online at http://github.com/ArionIfflandStettner/1A01). However, as shown in Fig. 3 and 4, the biomass composition of 1A01 varies based on growth rate. Therefore, after quantifying the GAM and NGAM, we investigated whether taking the GR dependence of the biomass composition of 1A01 into account (instead of applying the same glucose-derived biomass reaction to all conditions) would significantly improve the model’s predicted growth rates on carbon sources other than glucose. To wrap the GR dependence of the biomass composition of 1A01 into FBA (see Script S1 online at http://github.com/ArionIfflandStettner/1A01 for the full code), a growth rate on a given carbon source is first guessed and the corresponding biomass composition incorporated into the model via the biomass reaction. FBA then uses the model to predict an optimal growth rate. If this optimal growth rate matches the initially guessed growth rate, the script stops. Otherwise, the cycle repeats itself, with the FBA-predicted growth rate generating a new biomass composition (and, by extension, a new biomass reaction in the model), until the script converges (i.e., until the input and output growth rates match). We found that the script converges to approximately the same final growth rate, regardless of the initially guessed growth rate. For example, on galactose, it converges to a growth rate of about 0.27 h^−1^, regardless of whether one initially guesses 0.01 h^−1^ or 1 h^−1^. Comparing the results of GR-dependent FBA to “standard” FBA with a glucose-derived biomass reaction, both applied to growth on galactose, we found that the two gave very similar growth rates (see Fig. S7a in the supplemental material). The two methods gave larger differences in fluxes, with about half of the fluxes showing differences of at least 20% between glucose and galactose (Fig. S7b), reflecting substantially different dry mass compositions. Overall, even though the GR-dependent biomass composition can be accommodated, running FBA on the glucose-derived model provides reasonable approximations of both growth rates and flux distributions.

10.1128/msystems.00377-22.7 FIG S7 Here, we compare the output of FBA using the “standard” glucose-derived biomass reaction (gr_FBA_) and the GR-dependent biomass reaction (gr_GR-FBA_), when simulating growth of V. splendidus 1A01 on various carbon sources. (a) Bar plot of the percent difference between the growth rate output by the two FBA methods, for growth on pyruvate, galactose, and *N*-acetyl-glucosamine (GlcNAc). Note that the deviation between the two methods is the smallest for GlcNAc since the growth rates on GlcNAc and glucose are similar and so the biomass compositions are similar. (b) Histogram of the percent difference between the fluxes output by FBA using the “standard” glucose-derived biomass reaction and the fluxes output by FBA using the GR-dependent biomass reaction, when simulating the growth of V. splendidus 1A01 on galactose. Download FIG S7, PDF file, 0.04 MB. Copyright © 2023 Iffland-Stettner et al. 2023 Iffland-Stettner et al. https://creativecommons.org/licenses/by/4.0/ This content is distributed under the terms of the Creative Commons Attribution 4.0 International license.

**FIG 5:**
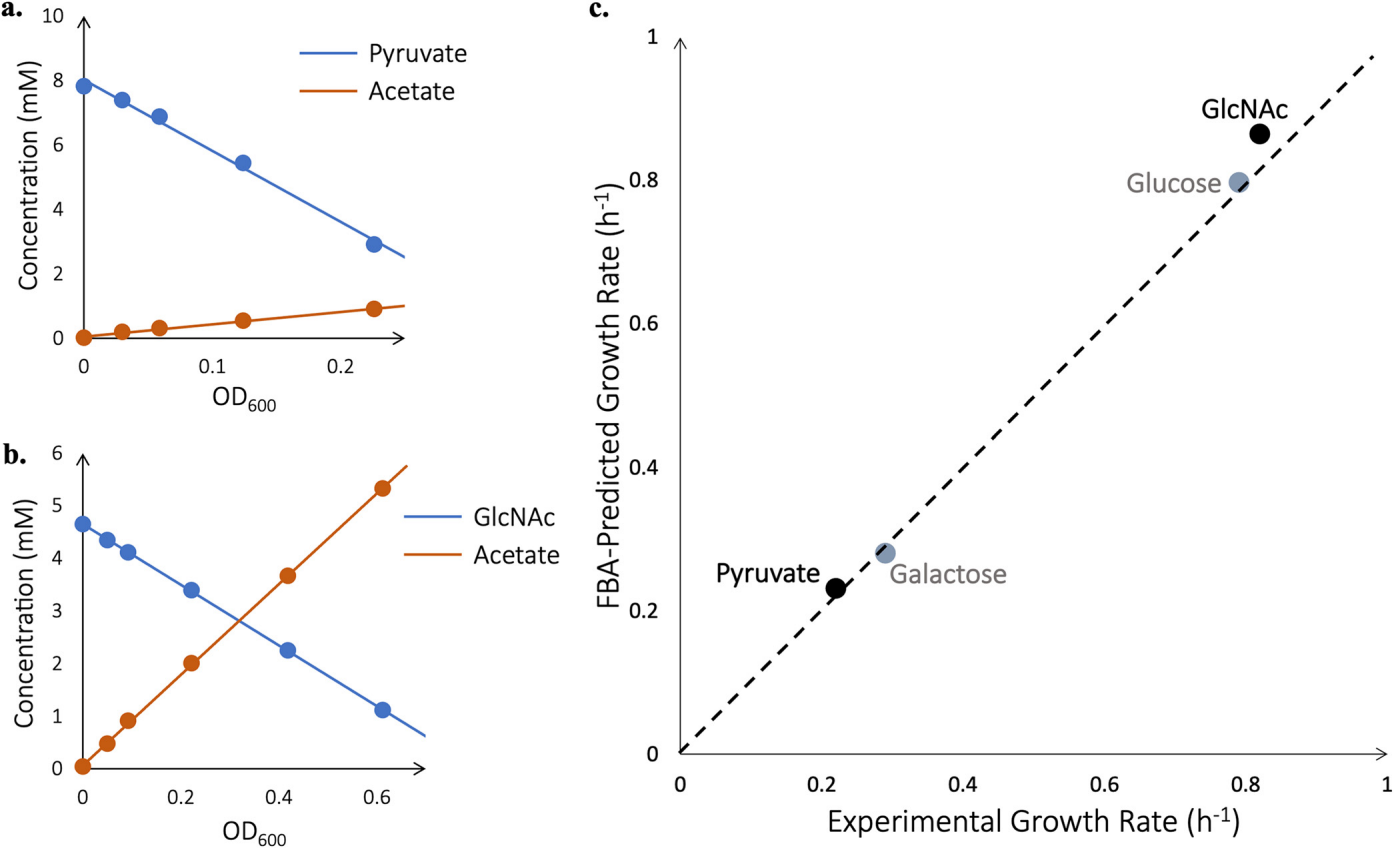
Quantitative testing of model predictions. (a and b) Shown in red is acetate accumulation, and shown in blue is pyruvate or *N*-acetylglucosamine (GlcNAc) depletion for a single batch culture of V. splendidus 1A01 (no replicates were performed). (c) Along the *x* axis are plotted experimentally measured growth rates of V. splendidus 1A01, and along the *y* axis are plotted theoretical growth rates predicted by the model. Shown in gray are substrates the model was trained on (glucose and galactose). Shown in black are substrates the model is being tested against (pyruvate and *N*-acetylglucosamine). The dashed line denotes perfect agreement between model and experiment. Note the model cannot be fit arbitrarily well to the training data, which is why the two points for glucose and galactose do not lie perfectly along the dashed line.

Next, the metabolic model was quantitatively tested against growth on carbon substrates other than those used to parameterize the model (i.e., glucose and galactose). This time, V. splendidus 1A01 was grown in batch on pyruvate and on *N*-acetylglucosamine. Again, the optical density, concentration of substrate, and concentration of acetate were measured at time intervals throughout exponential batch-culture growth (Fig. 5a and b). Rates of substrate consumption and acetate secretion were measured as before, but now they served as input into FBA rather than for inferring the parameters of the model (namely, the GAM and NGAM). More precisely, the model’s carbon uptake flux and acetate secretion flux were set to the observed values, and flux through the biomass reaction was optimized using FBA. For comparison, the same process was repeated for glucose and galactose. The good agreement between experiment and theory we observe for these last two substrates (as shown in Fig. 5c) is expected, given that the model was trained on them. However, we see the same agreement for pyruvate and, to a less extent, *N*-acetylglucosamine. (This imperfect agreement, in the case of *N*-acetylglucosamine, might be due to the coupling of carbon and nitrogen sources in this compound [38].) We conclude that the predictive power of the model extends beyond merely its training data. Still, the high-pressure liquid chromatography (HPLC) analysis in Fig. 4a and b and Fig. 5a and b was admittedly performed on single cultures of V. splendidus 1A01, without replicates. The absence of replicates prevents the calculation of a statistical measure of the model’s performance against experimental data, which future work will have to address.

## DISCUSSION

Despite its abovementioned shortcoming in terms of statistical testing (namely, the lack of batch-culture replicates), a GSMM was reconstructed in this work for V. splendidus 1A01, a chitin-degrading opportunistic pathogen in the ocean (5, 39). The model reconstruction required measuring a number of physiological parameters, which gave rise to the following technical challenges, partly due to the high salt concentration found in seawater (and in synthetic culture media that simulate seawater).

First, the GAM and NGAM are conventionally found by growing microbes in a chemostat (32). However, adapting wild organisms to long-term chemostat growth is often difficult (e.g., due to foaming, flocculation, and biofilm formation), and we opted for growing batch cultures (40–42) of V. splendidus 1A01 on different glycolytic carbon sources, at the maximal growth rate permitted by each carbon source (i.e., at saturating concentrations of the substrate). Because different carbon sources allow for different maximal growth rates, we were able to obtain a spread of growth rates that is obtained in a chemostat by tuning the dilution rate on a single carbon source. However, because strong overflow metabolism can occur at high growth rates (41), acetate excretion also had to be measured. This method of measuring the GAM and NGAM, while imperfect due to minor differences in metabolism due to differences associated with the specific substrates (e.g., glucose versus galactose), may be the most practical solution for wild organisms. For model organisms, the equivalence between metabolic parameters obtained from growth in batch culture and running a chemostat has been demonstrated (43).

Second, marine microbes differ in their biomass composition from other bacterial species, due at least in part to the high salt concentration in seawater (~0.3 to 0.4 M NaCl). For example, the RNA/protein ratio is reduced by ~25% when growing E. coli in ~0.3 to 0.4 M NaCl compared with growing it at its optimal osmolarity, although, given the growth-rate dependence of the RNA/protein ratio, this reduction in the ratio could be accounted for largely by the negative effect of salt on growth rate (44). However, high salt concentrations also lead to other physiological effects. Since all bacteria must produce osmolytes to balance external osmolarity, marine bacteria must produce more osmolytes, and this fact should be taken into account in their biomass composition, in order to arrive at a more accurate biomass reaction in the model. Determining the major osmolyte(s) employed by an organism is in principle nontrivial and would require quantitative metabolomics. In this case, we caught a break; we found the glutamate pool in V. splendidus 1A01 to be very high (~200 nmol/OD_600_/mL), over 10× times higher than the closely related glutamine pool and almost 50% higher than that in E. coli under similar osmolarity (~135 nmol/OD_600_/mL), as shown in Fig. S8 in the supplemental material. For E. coli (which has a cytoplasmic water content of ~2 μL/mg CDW (45), corresponding to ~1 μL/OD_600_/mL), the glutamate content (corresponding, again, to ~135 mM) together with the counterion potassium (of equal molarity) add up to ~270 mM osmolytes. This amount accounts for a large fraction of the external osmolarity imposed by salt, with the remainder balanced largely by the accumulation of trehalose (45). The amount of cytoplasmic water in V. splendidus 1A01 has not been measured. Assuming a similar amount of cytoplasmic water as in E. coli, the measured glutamate pool, together with the potassium counterion, would add up to ~400 mM osmolytes, which is similar to the external osmolarity of the medium. Our data thus suggest that glutamate is the sole major osmolyte used by V. splendidus 1A01 under growth conditions studied here.

10.1128/msystems.00377-22.8 FIG S8 Glutamate pools of E. coli NCM3722 (at 0.3 and 0.4 M NaCl in morpholinepropanesulfonic acid [MOPS] media [44]) and V. splendidus 1A01 (in 1 × SW, which corresponds to 0.37 M NaCl in the E. coli medium). Download FIG S8, PDF file, 0.04 MB. Copyright © 2023 Iffland-Stettner et al. 2023 Iffland-Stettner et al. https://creativecommons.org/licenses/by/4.0/ This content is distributed under the terms of the Creative Commons Attribution 4.0 International license.

Third, the high medium osmolarity made it challenging to measure the conversion factor of OD_600_ to CDW, which must be known to convert experimental measurements (most often conveniently done per culture volume, in units of per OD_600_*mL) to the unit of flux in FBA, mmol/gCDW/h, with gCDW corresponding to grams of CDW. To measure this conversion factor in a model organism like E. coli at normal osmolarity, the first step is to spin down a culture sample in a centrifuge and resuspend it in water, in order to wash away extracellular metabolites contained in the medium. However, if V. splendidus 1A01 is resuspended in water, a substantial fraction of cells burst due to its adaptation to the higher osmolarity in the sea, leading to a significant loss of biomass. We therefore had to develop a novel experimental technique (described in the Materials and Methods) to measure the CDW of a marine bacterium like V. splendidus 1A01.

In conclusion, we expect many of the technical issues we faced in building a model for V. splendidus 1A01 to reappear when building models for other marine microbes. Thus, we hope this work will not only shed light on the metabolic capabilities and behavior of V. splendidus 1A01 but also guide the reconstruction of GSMMs for the myriad other bacteria that populate our oceans.

## MATERIALS AND METHODS

### Culture conditions for quantitative measurements.

The growth media used were marine broth (Difco marine broth 2216) and a complete minimal medium for growing copiotrophic, heterotrophic marine bacteria. Marine broth was prepared by dissolving 37.4 g/L in double-distilled water (ddH_2_O), boiling for 1 min, and filtering through a 0.22-μm filter for sterilization. It was stored at room temperature. The minimal medium consisted of a carbon source, 10 mM NH_4_Cl, 0.5 mM Na_2_HPO_4_, 1 mM Na_2_SO_4_, simple salts to mimic seawater (0.343 M NaCl, 14.75 mM MgCl_2_ · 6H_2_O, 4 mM CaCl_2_ · 2H_2_O, and 27 mM KCl, which is designated 1× SW), 40 mM HEPES (pH 8) as the buffer, and trace metals such as iron. All components were filter sterilized using a 0.22-μm filter. The minimal medium was stored at 4°C. For a full description of the preparation and composition of the minimal medium, see reference 46.

Preparing batch cultures used for measuring RNA, protein, metabolites, and cell dry weight of 1A01 involved three steps, as follows: (i) a seed culture, (ii) a preculture, and (iii) an experimental culture. The seed culture was started by inoculating 2 mL of marine broth in a 16- by 125-mm test tube (borosilicate glass; Fisherbrand, cat. no. 14-961-30) from a single colony on a marine broth/agar plate. Once the seed culture saturated (which took ~7 h), the cells were washed and resuspended in 1× SW to an OD_600_ of ~1 before being diluted into the preculture with experimental medium (3 mL in a 18- by 150-mm tube) for growth overnight, such that, by the following day, the preculture doubled ≥10 times and remained growing exponentially. While the preculture was still in exponential growth, we diluted it into fresh experimental medium prewarmed to 27°C to an OD_600_ of 0.01 to 0.02 to start the experimental culture. This culture was allowed to grow for several doublings before samples were taken for quantitative measurements. Altogether, this growth protocol ensures that each culture was grown continuously for 5 to 7 doublings under the same condition by the time of measurements (see reference 46 for details). All cultures (seed, preculture, and experimental) were grown in a water bath shaker at 27°C with shaking at 250 rpm. OD_600_ was measured using a Thermo Scientific Genesys 20 or 30 spectrophotometer calibrated to the same standard. Growth rates were determined by linear regression from exponential-phase growth curves, as shown in Fig. 2a.

### Culture conditions for growth phenotype screening.

High-throughput screening was also performed to obtain a coarse growth phenotype for V. splendidus 1A01. A frozen culture sample (5% dimethyl sulfoxide [DMSO]) was thawed (at room temperature), then 20 μL of the stock culture was transferred to 180 μL marine broth in 96-well plates, and the culture was grown for 94 h at room temperature without shaking. The cultures were diluted 1:1 in carbon-free minimal medium (see Text S1 in the supplemental material for the full recipe) for 2 h and then transferred into 384-well plates filled with 70 μL minimal medium per well and 1 of 78 added carbon sources (see Table S1 online at http://github.com/ArionIfflandStettner/1A01 for full list) using a pinning tool (V&P Scientific VP 408; 0.2-μL hanging drop volume). The concentration of carbon atoms was normalized to 40 mM for each carbon source. All plates were then covered with transparent film (Life Technologies MicroAmp Optical Adhesive Film) and stored in the dark at room temperature. All plates were read at least once a day for 10 days using a stack plate reader (Tecan Spark) to measure optical density at 600 nm.

10.1128/msystems.00377-22.9 TEXT S1 Minimal MBL medium. Download Text S1, PDF file, 0.4 MB. Copyright © 2023 Iffland-Stettner et al. 2023 Iffland-Stettner et al. https://creativecommons.org/licenses/by/4.0/ This content is distributed under the terms of the Creative Commons Attribution 4.0 International license.

### RNA, protein, and metabolite measurements.

RNA and proteins were measured as described before (40), with modifications. Since the cells from cultures grown on glucose, *N*-acetylglucosamine (GlcNAc), and glucosamine were not harvested well by the short centrifugation employed in the regular protocol, they were first chilled on ice-water for 5 min and harvested by centrifugation at 15,000 rpm (Eppendorf Centrifuge 5424) for 15 to 20 min at 4°C. For protein measurements, the cells were further rinsed in the minimal medium lacking carbon, nitrogen, and phosphorus sources and harvested by centrifugation for 15 to 20 min. The effects of the long centrifugation at 4°C were assessed by comparing measured protein concentrations with those using the regular short centrifugation for cells grown on glycerol since glycerol cultures were harvested well by the regular short centrifugation. Little difference (<2%) was observed.

For RNA measurements, 1.5 mL of an exponentially growing culture was pelleted, fast frozen on dry ice, and stored. Pellets were thawed, washed twice with 0.7 M cold HClO_4_, and then digested for 60 min at 37°C using 300 μL of 0.3 M KOH. Samples were stirred periodically. The cell extracts were then neutralized with 100 μL of 3 M HClO_4_ and centrifuged at 13,000 rpm for 3 min. The soluble fraction was collected and the remaining pellets washed twice with 550 μL of 0.5 M HClO_4_. The resulting final volume of 1.5 mL was centrifuged once more to eliminate remaining debris, and its absorbance at 260 nm was measured using a Bio-Rad spectrophotometer. The RNA concentration was determined as OD_260_*31/OD_600_, where the conversion factor is based on RNA’s extinction coefficient (40).

Protein amounts were quantified using the Biuret method. A total of 1.5 mL of an exponentially growing culture was pelleted, washed with 1× SW, resuspended in 200 μL of 1× SW, and fast frozen on dry ice. The cell pellet was then thawed at room temperature. Next, 100 μL of 3 M NaOH was added to the pellet, and samples were incubated on a heat block at 100°C for 5 min to hydrolyze the proteins. Protein amounts in the samples were determined using the Biuret method. A total of 100 μL of 1.6% CuSO_4_ was added to the protein extracts, and samples were centrifuged for 3 min at 13,000 rpm. The absorbance of the soluble fraction was read at OD_555_ using a spectrophotometer. A series of 200 μL bovine serum albumin (BSA) standards were taken through the same procedure to get a standard curve.

Glutamate and glutamine pools in minimal medium were measured by HPLC with the no-harvest protocol as described previously (47–49). Since glutamate and glutamine were also found in the medium, the intracellular glutamate or glutamine pool was obtained by subtracting the amount of glutamate or glutamine in the medium from that in the whole culture. The amount of glutamate in the medium depends on OD_600_ and ranges between 5 and 16 μM on glucose and GlcNAc, which corresponds to 10 to 20% of the whole culture, The amount of glutamine also depends on the OD_600_ and ranges between 0.4 and 1.1 μM, which corresponds to 50 to 90% of the whole culture grown on galactose. Extracellular carbohydrates were measured by HPLC using a refractive index column as described in reference 46.

According to its genome, V. splendidus 1A01 also has the capability to produce carbon storage compounds, such as polyhydroxybutyrate (PHB). However, the amount of carbon storage compounds in a culture of V. splendidus 1A01 was not measured in this study. Therefore, carbon storage compounds were excluded from the biomass reaction of the model.

### Cell dry weight measurements.

In a regular protocol to measure cell dry weight (CDW), a cell pellet is washed with water to remove salts from the culture medium. However, such a protocol cannot be applied to bacterial cells grown at high osmolarity because a substantial fraction of the cells are lysed in water. Therefore, we had to develop a novel protocol to estimate the CDW of bacterial cells grown at high osmolarity.

A V. splendidus 1A01 culture was grown to an OD_600_ of 0.5, chilled on ice-cold water for 5 to 15 min, and harvested by centrifugation at 15,000 rpm (Eppendorf Centrifuge 5424) at 4°C. The cell pellet was washed with the same volume of cold NaCl solution as the culture and washed again with 40 mL of the same NaCl solution. Concentrations of NaCl were 0.45 M for cultures grown at 1× SW and 0.52 M for cultures grown at 1.25× SW or in marine broth. The density of the washing NaCl solution was measured with water as a reference. After the wet pellet was weighed, it was suspended in water, transferred to a weighing dish, and dried in an oven at 85 to 95°C until the weight became stable, typically for 3 days. The dry pellet was weighed within 10 s before it absorbed water and thereby significantly increased in weight.

CDW was estimated as follows. Let *x* and *y* be the weights of dry and wet cell pellets, respectively; *ρ* the weight of NaCl per the weight of water in the washing solution; *w* the harvested amount of cells in units of OD_600_·mL; and *z* the weight of extracellular water in the wet pellet. With these parameters, we can represent *α*, the CDW per OD_600_·mL, as a function of *β*, the cellular water weight per CDW.

From the equality of CDW,

(1) αw=x−ρz

From the equality of water weight present in the wet pellet,

(2) z=y−x−βαw

From the above two equations, we obtain

(3) $$\alpha =\frac{x\left(1+\rho \right)-\rho y}{w(1-\rho \beta )}$$

In E. coli, *β* is reported to be ~2 mg/mg CDW (45). Like E. coli, V. splendidus 1A01 is a rod-shaped, Gram-negative bacterium. Hence, we assumed β=2 ± 1 mg cellular water/mg CDW for V. splendidus 1A01. The difference between *x* and *y* sets the upper bound on the weight of cellular water in a wet pellet, $x\beta_{\max}$, which leads to $\beta <\;\beta_{\max}=\frac{y}{x}\;-\;1$. If the supernatant after washing was carefully removed, $\beta_{\max}$ is typically below 5. Within $0\;<\;\beta \;<\;\beta_{\max}\;\sim 5,\;\alpha$ is only weakly dependent on *β* (Fig. S1), and hence, robust to error in the estimation of *β* within this range. This weak dependence results from the much lower density of the NaCl washing solution than the cellular mass density (ρ=0.0306 for 0.52 M NaCl washing solution compared to ~0.5 mg CDW/mg cellular water).

10.1128/msystems.00377-22.1 FIG S1 An example of CDW per OD_600_·mL, *α*, as a function of cellular water weight per CDW, *β*. The culture was grown in a minimal medium supplemented with 10 mM glucose at 1.25 × SW. It can be seen that *α* is only weakly dependent on *β*. With β=2 ± 1 mg cellular water/mg CDW, *α* is estimated to be 0.536 ± 0.015 mg per OD_600_·mL. Download FIG S1, PDF file, 0.01 MB. Copyright © 2023 Iffland-Stettner et al. 2023 Iffland-Stettner et al. https://creativecommons.org/licenses/by/4.0/ This content is distributed under the terms of the Creative Commons Attribution 4.0 International license.

Equation 3 can also be rewritten as follows:

(4) $$y=\left(\rho^{-1}+1\right)\left\{x-\frac{(1-\rho \beta )w\alpha }{1+\rho }\right\}$$

Equation 4 predicts that a linear relation is obtained when *y* is plotted against *x* and that *ρ* and *α* can be estimated from its slope and *x*-intercept, respectively. To test this prediction, we dispensed the culture grown at 1.25 × SW on glucose into three aliquots of the same volume and obtained three washed wet pellets. Then we added back 0, 100, and 200 mL of the washing solution to each wet cell pellet and measured *x* and *y* for each pellet. As predicted, *x* and *y* showed a linear relation (R^2^ = 0.9991, Fig. S2). ρ=0.0298 was estimated from the slope, which is close to the measured value (ρ=0.0306). α=0.550 was estimated from the *x*-intercept with β=2, which is also close to those estimated from Equation 3 for the three pellets (α=0.547 ± 0.001). This result also demonstrates that the direct estimate from Equation 3 is precise enough. The CDW of V. splendidus 1A01 growing in a number of different media was estimated in this way using Equation 3 and is shown in Fig. 2b.

10.1128/msystems.00377-22.2 FIG S2 The linear relation between the weights of wet and dry cell pellets. Cells were grown in minimal media at 1.25 × SW supplemented with 10 mM glucose. Download FIG S2, PDF file, 0.02 MB. Copyright © 2023 Iffland-Stettner et al. 2023 Iffland-Stettner et al. https://creativecommons.org/licenses/by/4.0/ This content is distributed under the terms of the Creative Commons Attribution 4.0 International license.

### Constructing a model of biomass composition.

V. splendidus 1A01 was grown in marine broth and in minimal medium on glucose, glucosamine, glycerol, and galactose. RNA, protein, and CDW were quantified in each culture (in units of mg/OD_600_/mL) and plotted against the growth rate, yielding the linear relations shown in Fig. 2b. For the model, RNA and protein measurements corresponding to growth on glucose were used to build the biomass reaction (after converting from mg/OD_600_/mL to mg/mg CDW using the mg CDW/OD_600_/mL for glucose). These measurements quantify the total RNA and protein in a biomass sample, not the relative proportions of the monomers that make up RNA and protein. As an approximation frequently made in the FBA literature (32), the relative proportions of the 20 amino acids that make up protein were inferred from their relative proportions in the protein-coding regions of the genome of V. splendidus 1A01. Likewise, the relative proportions of the 4 nucleotides that make up RNA were inferred from their relative proportions in the rRNA-coding regions of the V. splendidus 1A01 genome (rRNA makes up the bulk of RNA in bacterial cells [50]). The major osmolyte (glutamate) was quantified at the standard osmolarity of 1 × SW on glucose (10 mM), galactose (10 mM), glycerol (20 mM), and GlcN (20 mM), and the results are shown in Fig. 2b. Intracellular glutamine, which was measured as a comparative reference, was found at much lower levels (see Table S2 online at http://github.com/ArionIfflandStettner/1A01) but was also included in the model. Like with RNA and protein, osmolyte measurements corresponding to growth on glucose (see Table S2 online at http://github.com/ArionIfflandStettner/1A01) were used to build the biomass reaction of the model. The unquantified components of biomass (namely, DNA, lipopolysaccharides [LPS], lipids, murein, inorganic ions, and soluble pools) reflect the E. coli model iAF1260 (37). Because they represent 20% of E. coli biomass and only 17% of V. splendidus 1A01, the total fraction of biomass these unquantified components represent was scaled down for V. splendidus 1A01, while keeping their relative proportions the same as those in E. coli. As with RNA and protein, the relative proportions of the 4 nucleotides that make up DNA were inferred from their relative proportions in the V. splendidus 1A01 draft genome. Since populating parts of the biomass reaction of 1A01 with coefficients from E. coli is an approximation, a sensitivity analysis of the model performance to slight deviations from E. coli (see Fig. S3) was carried out. In this analysis, for every unquantified component of biomass (DNA, LPS, lipids, murein, inorganic ions, or soluble pools), the fraction of biomass it represents was raised or lowered arbitrarily by up to 25%, which led to only negligible changes in flux through the biomass reaction (less than 1.4%). The complete biomass reaction is shown in Table S3 (online at http://github.com/ArionIfflandStettner/1A01), along with all calculations that lead from the above measurements and approximations to the final biomass reaction.

Although the model biomass reaction reflects the biomass composition of V. splendidus 1A01 grown on glucose, the biomass composition for cells grown in other media varies based on growth rate (GR), as shown in Fig. 2b. We therefore wrote a MATLAB script (see Script S1 online at http://github.com/ArionIfflandStettner/1A01) to incorporate the GR-dependent biomass composition of 1A01 in FBA, using the linear fits of RNA, protein, and CDW content given in Fig. 2b.

### Reconstruction and gap-filling of draft metabolic model.

Estimated to be 99.4% complete by CheckM (51, 52) (using the g__Vibrio marker gene set; the missing marker genes being PF07219, HemY protein N terminus; TIGR01389, ATP-dependent helicase RecQ; TIGR02195, lipopolysaccharide heptosyltransferase II; and TIGR02143, tRNA [(uracil(54)-C(5))-methyltransferase]), the annotated genome of V. splendidus 1A01 was uploaded to BioCyc, where it was reconstructed into a draft metabolic model (in the form of an SBML file) using Pathway Tools (53). The reactions and metabolites of the model therefore conform to BioCyc nomenclature (except where reactions from the BiGG (54) database were inserted to fill gaps in metabolic pathways). The SBML file was then imported into MATLAB for model curation (32), which included the following gap-filling. If V. splendidus 1A01 grew on a given carbon source *in vivo* (growth being defined as achieving an OD_600_ increase of at least 0.9 after 20 h), the minimum number of reactions were added to the model that enabled flux through its biomass reaction on the same carbon source. Exchange reactions were added to the model only for such experimentally verified carbon sources.

### Flux balance analysis.

Flux balance analysis (FBA) is a widely adopted computational method to model cellular metabolism (10). When applied to a GSMM, FBA predicts the steady-state distribution of fluxes that optimizes a certain objective function. In this work, to simulate bacterial growth, biomass production was chosen as the objective function. To calculate the NGAM, ATP production was chosen as the objective function (see “Estimation of GAM and NGAM”). Briefly, FBA is implemented as a linear programming problem and formulated in matrix notation as follows:

(5) $$\begin{array}{c} \mathrm{maximize}\;Z=c^{T}v\\ \mathrm{subject}\;\mathrm{to}\;\mathrm{Sv}=0\\ \mathrm{and}\;v_{\min}\;\leq \;v\;\leq \;v_{\max} \end{array}$$

where *Z* denotes the objective function, *c* the relative weight of every reaction in the objective function, *v* the flux distribution, *S* the stoichiometric matrix, *v*_min_ the lower bounds on metabolic fluxes, and *v*_max_ the upper bounds. We performed FBA in MATLAB using the Gurobi optimizer (55). A simple script for performing FBA with the V. splendidus 1A01 model is provided (see Script S2 online at https://github.com/ArionIfflandStettner/1A01).

### Estimation of GAM and NGAM.

To determine the GAM and NGAM of V. splendidus 1A01, we measured batch-culture growth rate and net carbon influx on glucose and galactose (Fig. 4c). For a detailed description of growth medium, measurement and calculation of growth rate, as well as measurement and calculation of substrate intake and organic acid excretion, please see reference 46. During constant exponential growth on glucose in batch culture, V. splendidus 1A01 excreted acetate at a constant rate (a previous study [46] found no other significantly excreted hydrocarbon). The rate of acetate accumulation was subtracted from the rate of substrate depletion to calculate the net carbon influx (or carbon utilization rate). By plotting carbon utilization rate against growth rate and taking the y-intercept of the line (see Fig. 4c), we obtained the baseline rate of carbon utilization by V. splendidus 1A01 in the absence of biomass production. We then imposed this carbon utilization rate in FBA (by manipulating upper and lower bounds on substrate uptake and acetate secretion fluxes) and maximized ATP production (while setting flux through the biomass reaction to zero) to arrive at the value of the NGAM, which in the model is its own ATP hydrolysis reaction (ATP + H_2_O → ADP + Pi). The GAM (which can be found in Table S3 online at http://github.com/ArionIfflandStettner/1A01, as part of the complete biomass reaction of the model) was also calculated using FBA, by fitting the model’s performance to the line in Fig. 4c. More precisely, we imposed the observed carbon utilization rates in FBA (again, by manipulating upper and lower bounds on substrate uptake and acetate secretion fluxes) and increased the GAM until FBA-predicted growth rates matched experimental values.

### Model validation.

A metabolic model must be validated against experimentally measured growth on substrates other than those used to parameterize the model (in this work, 5 mM glucose and 5 mM galactose). Therefore, batch cultures of V. splendidus 1A01 were grown on 10 mM pyruvate and 5 mM *N*-acetylglucosamine (the monomer of chitin, on which particles 1A01 was first isolated [5]), while, again, tracking growth rate, substrate depletion, and acetate accumulation (Table S4 online at http://github.com/ArionIfflandStettner/1A01). Experimentally measured rates of substrate uptake and acetate secretion were then used, in FBA, to constrain the corresponding fluxes in the model to their observed values, before optimizing flux through the biomass reaction, thereby obtaining theoretically optimal growth rates, which, for model validation, were compared to experimentally measured growth rates.

### Software availability.

The genome-scale metabolic model of V. splendidus 1A01, as well as the code to run FBA (Script S2) and GR-dependent FBA (Script S1) on this model, are available in a GitHub repository online at https://github.com/ArionIfflandStettner/1A01.
