# Supplementary figures and images for: A Genome-Scale Metabolic Model of Marine Heterotroph Vibrio splendidus Strain 1A01
Source: mSystems. 2023 Feb 28;8(2):e00377-22. doi: 10.1128/msystems.00377-22 (PMC10134806; doi:10.1128/msystems.00377-22)

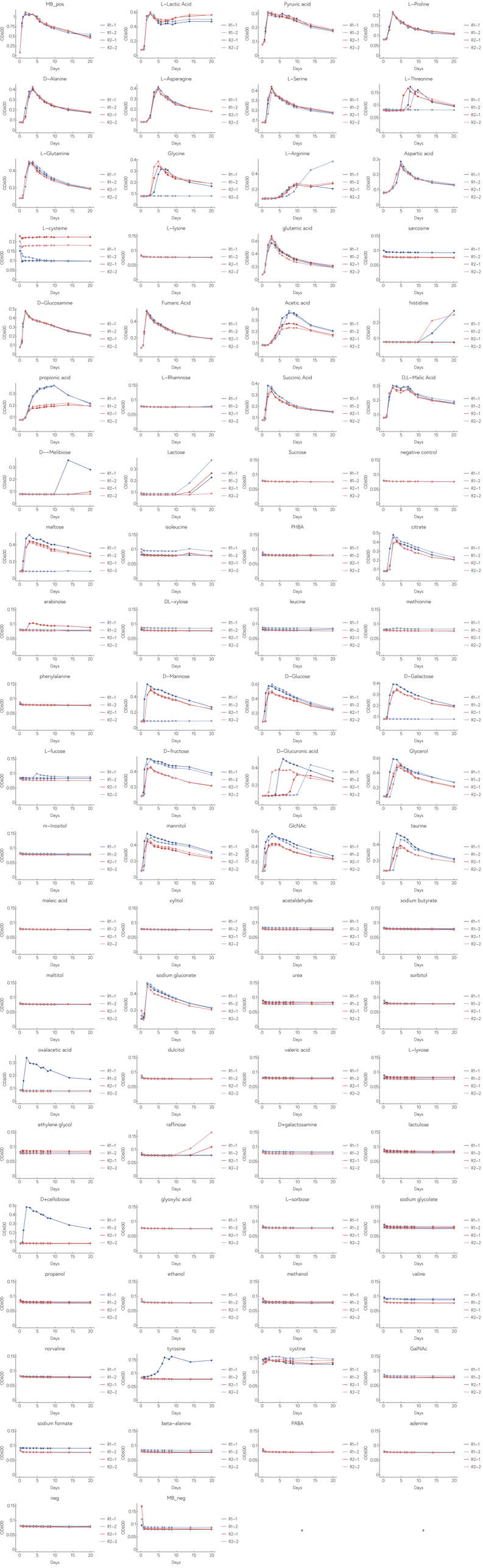

Supplement: FIG S4 [file msystems.00377-22-s0005.pdf]

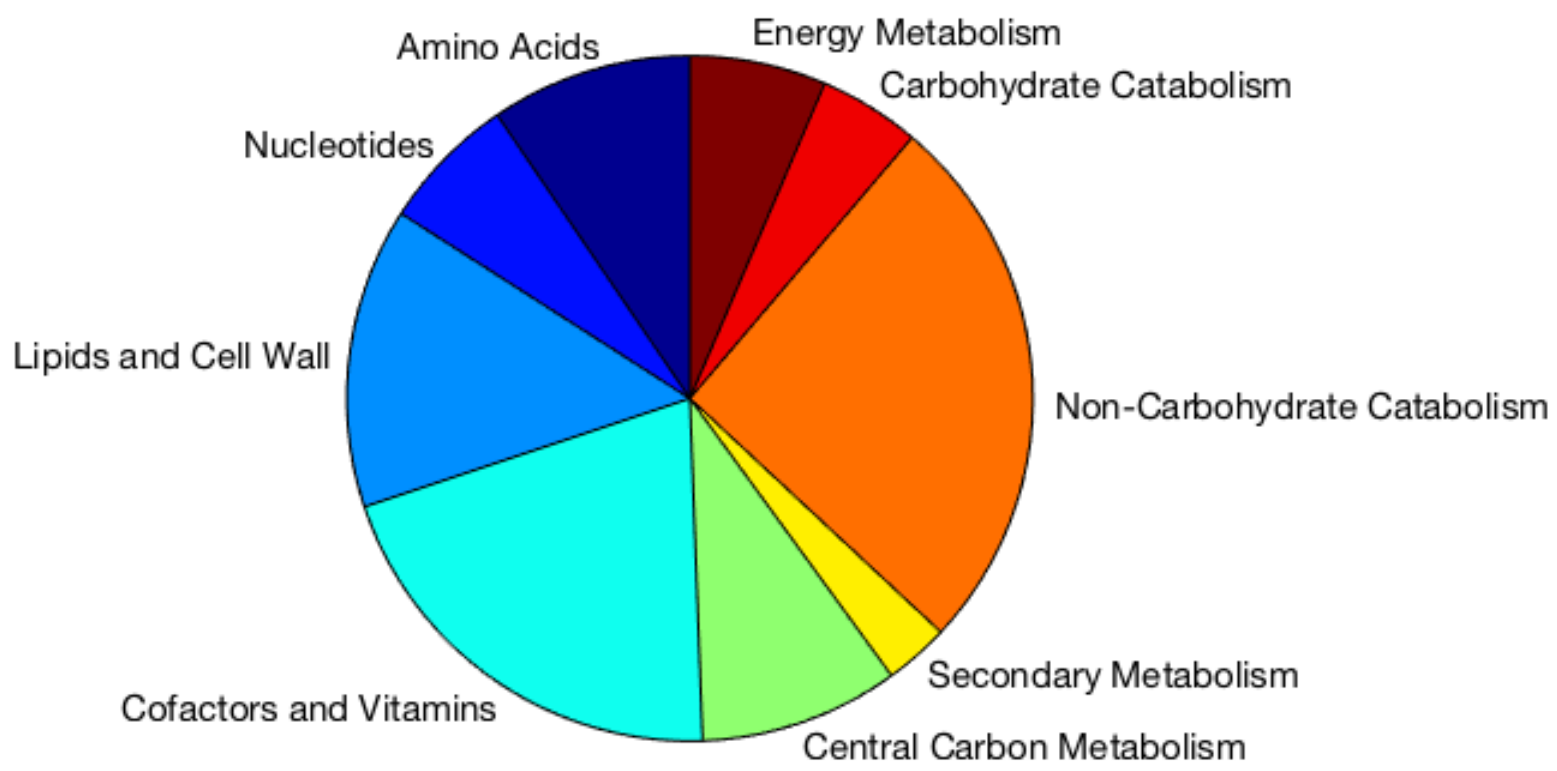

Supplement: FIG S5 [file msystems.00377-22-s0006.pdf]

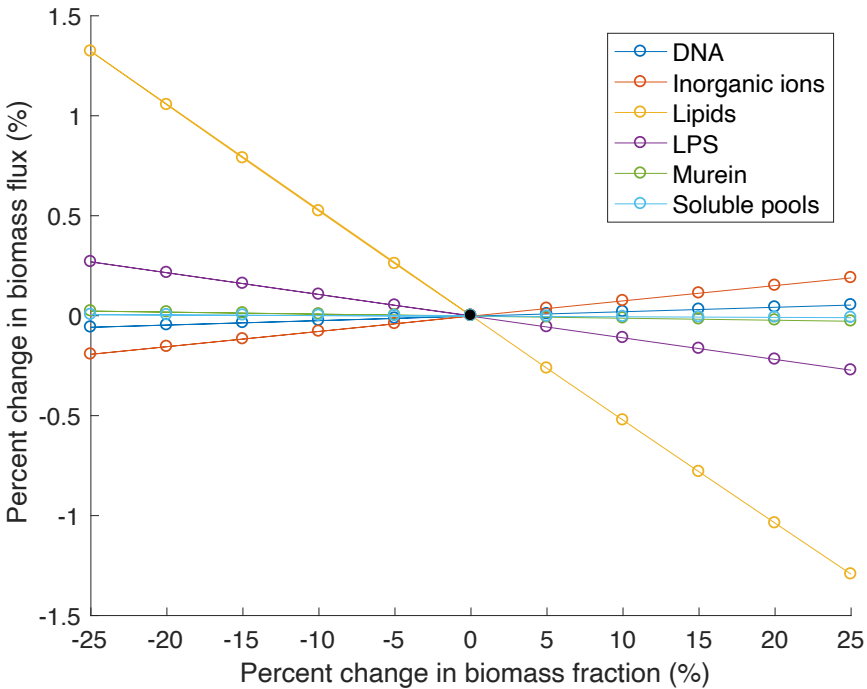

Supplement: FIG S3 [file msystems.00377-22-s0004.pdf]

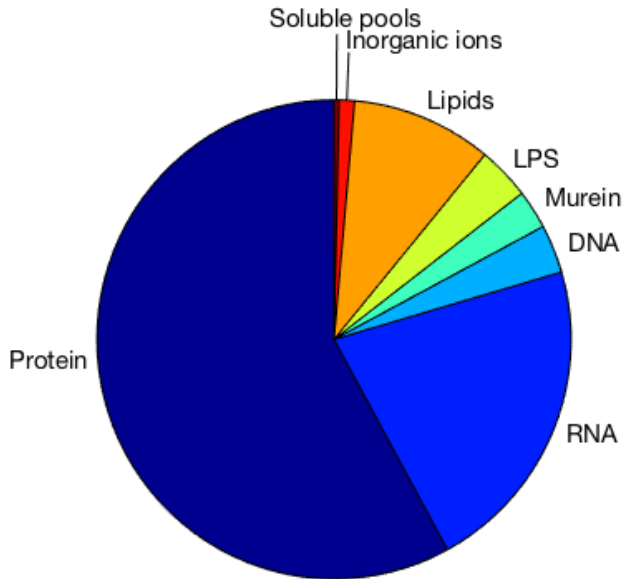

Supplement: FIG S6 [file msystems.00377-22-s0009.pdf]

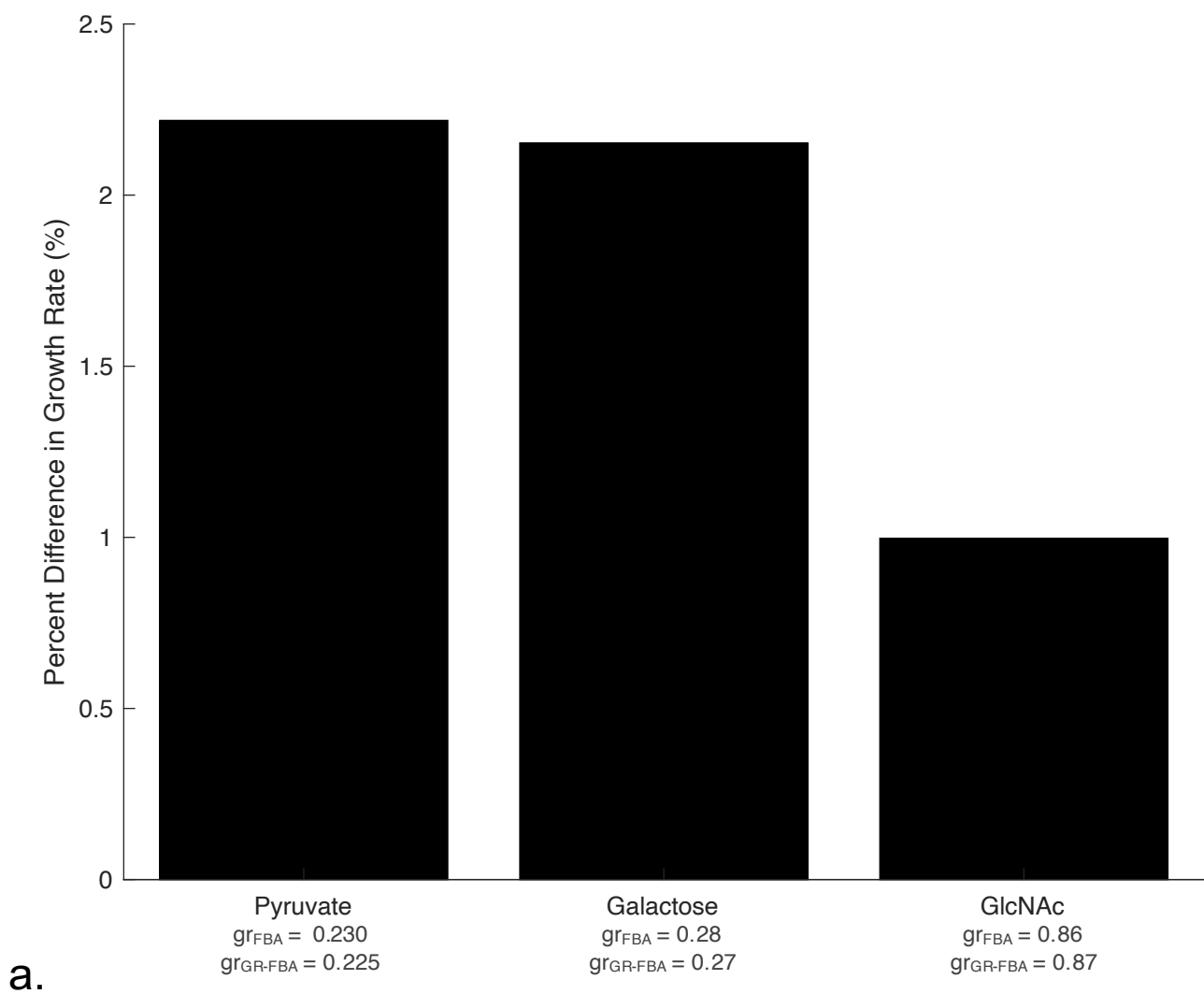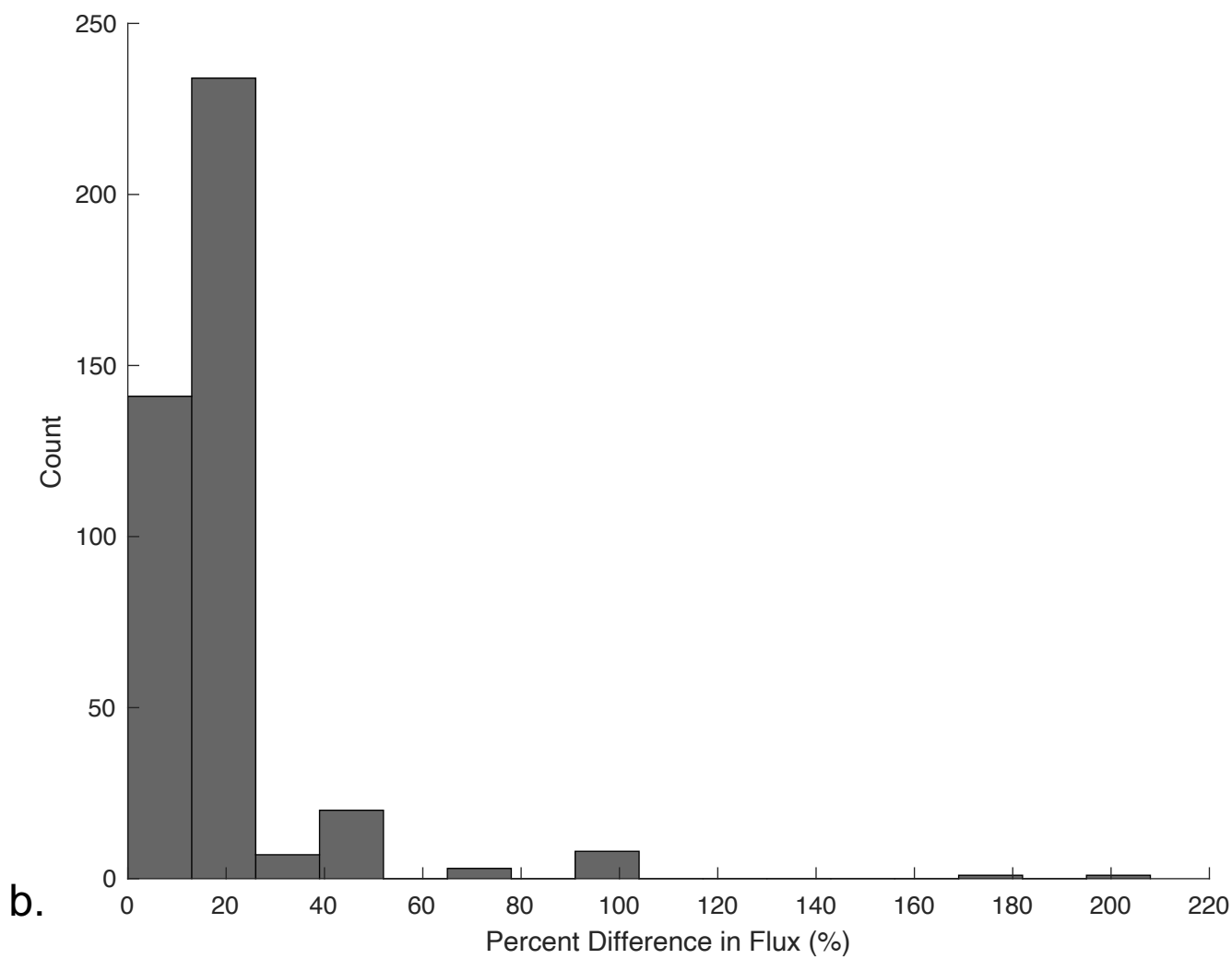

Supplement: FIG S7 [file msystems.00377-22-s0007.pdf]

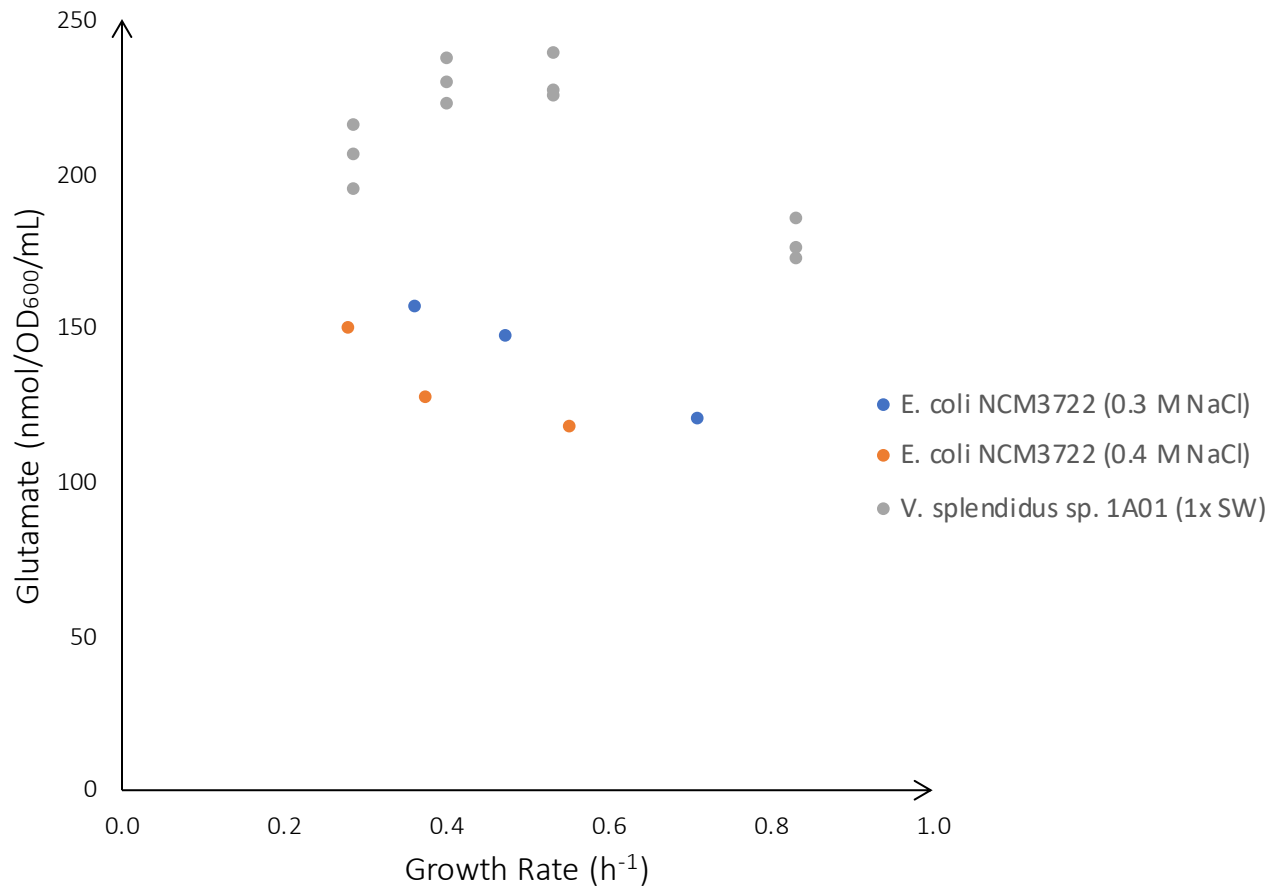

Supplement: FIG S8 [file msystems.00377-22-s0008.pdf]

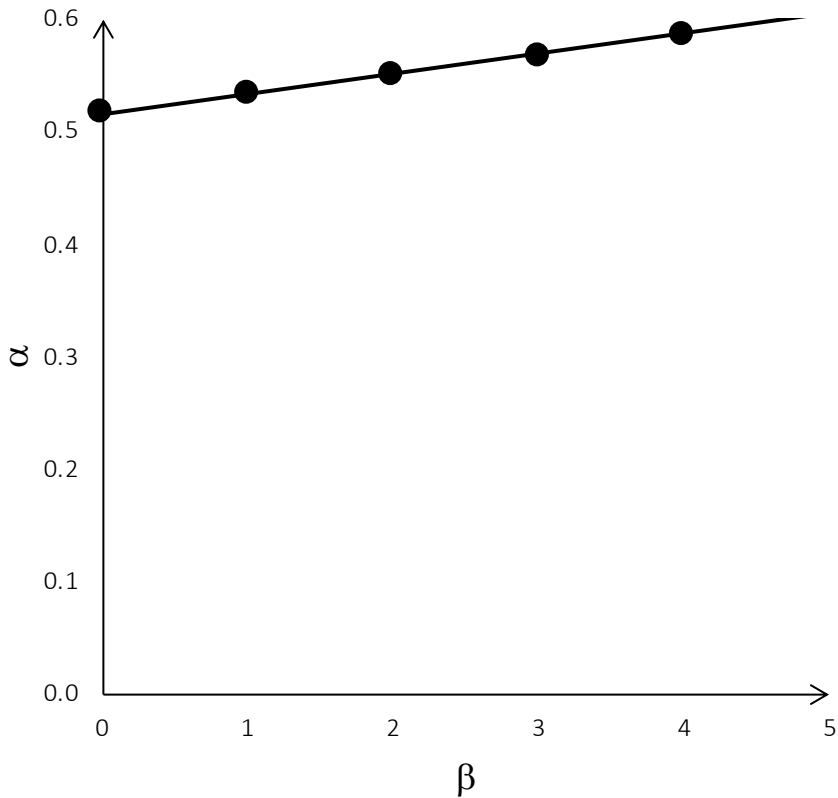

Supplement: FIG S1 [file msystems.00377-22-s0002.pdf]

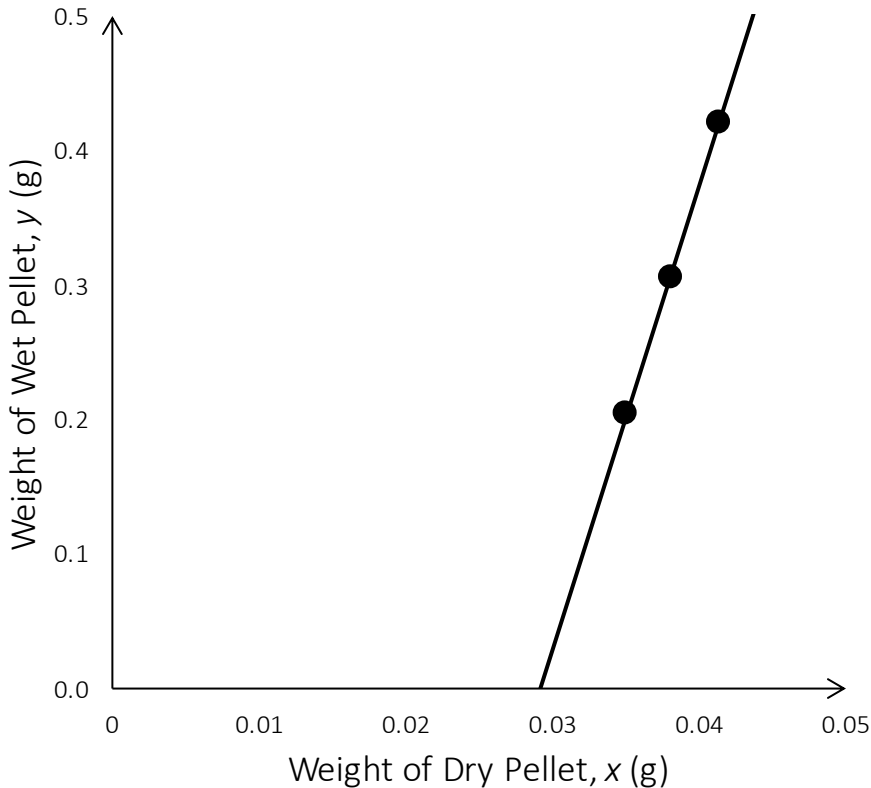

Supplement: FIG S2 [file msystems.00377-22-s0003.pdf]
