# Supplementary material for: A Genome-Scale Metabolic Model of Marine Heterotroph Vibrio splendidus Strain 1A01
Source: mSystems. 2023 Feb 28;8(2):e00377-22. doi: 10.1128/msystems.00377-22 (PMC10134806; doi:10.1128/msystems.00377-22)
Supplement: TEXT S1 [file msystems.00377-22-s0001.pdf]

Document S1: Minimal MBL Medium

4x Seawater

This is the base for all defined media.

Component	Amount (per L)	FW (g/mol)	Concentration (mM)
NaCl	80 g	58.44	1369
$MgCl_2$-6H_2O	12 g	203.20	59
$CaCl_2$-2H_2O	0.60 g	147.02	4
KCl	2.0 g	74.56	27

Make 1 L and filter sterilize through 0.2 µm.

1000x Trace Minerals (per L)

Add to recapitulate the ionic composition of seawater.

Dissolve in 20 mM HCl (to avoid precipitate):

Substance	mg / L
$FeSO_4$ * 7H_2O	2100
H_3BO_3	30
$MnCl_2$ * 4H_2O	100
$CoCl_2$ * 6H_2O	190
$NiCl_2$ * 6H_2O	24
$CuCl_2$ * 2H_2O	2
$ZnSO_4$ * 7H_2O	144
Na_2MoO_4 * 2H_2O	36
$NaVO_3$	25
$NaWO_4$ 2H_2O	25
Na_2SeO_3 5H_2O	6

**Note*: $NaVO_3$ and $NaWO_4$ * 2H_2O should only be opened in the hood.

Filter sterilize through 0.2 µm.
Store at 4 C in the dark. Stable for a couple months.
For long-term storage, freeze at -20 C in aliquots of 1 mL.

1000x Vitamins

Dissolve in 10 mM MOPS, pH 7.2:

Substance	mg/L
Riboflavin	100
D-Biotin	30
Thiamine hydrochloride	100
L-ascorbic acid	100
Ca-d- pantothenate	100
Folate	100
Nicotinate	100
4-aminobenzoic acid	100
pyridoxine HCl	100
Lipoic acid	100

NAD	100
Thiamin pyrophosphate	100
Cyanocobalamin	10

Titrate with a couple of drops of 5 M NaOH to avoid precipitate.
Filter sterilize through 0.2 µm.
Store at 4 C in the dark. Stable for a couple months.
For long-term storage, freeze at -20 C in 1 mL aliquots.

Nitrogen source
1 M ammonium chloride (100x)

Dissolve 2.14 g NH_4Cl in 40 mL distilled water.
Filter sterilize through 0.2 µm.

Phosphorus source
0.5 M phosphate dibasic (500x)

Dissolve 2.84 g Na_2HPO_4 in 40 mL distilled water.
Filter sterilize through 0.2 µm.

Sulfur source
1 M sodium sulfate (1000x)

Dissolve 5.68 g Na_2SO_4 in 40 mL distilled water.
Filter sterilize through 0.2 µm.

HEPES buffer:
1 M HEPES buffer (20x), pH 8.2

Per L
260.29 g HEPES sodium salt
Dissolve in 750 mL distilled water. Adjust pH to 8.2 with concentrated HCl and constant stirring.
Bring final volume to 1 L with water. Filter sterilize through 0.2 µm. Store at 4 C.

Basic recipe for medium (40 mL)

0.04 mL vitamins
0.04 mL trace metals
0.04 mL 1000x sodium sulfate
0.08 mL of 500x phosphate dibasic
0.4 mL of 100x ammonium chloride

2 mL of 20x GlcNAc
2 mL of 20x HEPES buffer, pH 8.2
10 mL of 4x seawater
25.4 mL of autoclaved ddH_2O
